# Fabrication of a pediatric torso phantom with multiple tissues represented using a dual nozzle thermoplastic 3D printer

**DOI:** 10.1002/acm2.13064

**Published:** 2020-10-19

**Authors:** Matthew M. Mille, Keith T. Griffin, Roberto Maass‐Moreno, Choonsik Lee

**Affiliations:** ^1^ Division of Cancer Epidemiology and Genetics National Cancer Institute National Institutes of Health Rockville MD USA; ^2^ Department of Nuclear Medicine National Institutes of Health Bethesda MD USA

**Keywords:** 3D printing, computed tomography, computer‐aided design, physical phantoms, tissue simulation

## Abstract

**Purpose:**

To demonstrate an on‐demand and nearly automatic method for fabricating tissue‐equivalent physical anthropomorphic phantoms for imaging and dosimetry applications using a dual nozzle thermoplastic three‐dimensional (3D) printer and two types of plastic.

**Methods:**

Two 3D printing plastics were investigated: (a) Normal polylactic acid (PLA) as a soft tissue simulant and (b) Iron PLA (PLA‐Fe), a composite of PLA and iron powder, as a bone simulant. The plastics and geometry of a 1‐yr‐old computational phantom were combined with a dual extrusion 3D printer to fabricate an anthropomorphic imaging phantom. The volumetric fill density of the 3D‐printed parts was varied to approximate tissues of different radiographic density using a calibration curve relating the printer infill density setting to measured CT number. As a demonstration of our method we printed a 10 cm axial cross‐section of the computational phantom’s torso at full scale. We imaged the phantom on a CT scanner and compared HU values to those of a 1‐yr‐old patient and a commercial 5‐yr‐old physical phantom.

**Results:**

The phantom was printed in six parts over the course of a week. The printed phantom included 30 separate anatomical regions including soft tissue remainder, lungs (left and right), heart, esophagus, rib cage (left and right ribs 1 to 10), clavicles (left and right), scapulae (left and right), thoracic vertebrae (one solid object defining thoracic vertebrae T1 to T9). CT scanning of the phantom showed five distinct radiographic regions (heart, lung, soft tissue remainder, bone, and air cavity) despite using only two types of plastic. The 3D‐printed phantom demonstrated excellent similarity to commercially available phantoms, although key limitations in the printer and printing materials leave opportunity for improvement.

**Conclusion:**

Patient‐specific anthropomorphic phantoms can be 3D printed and assembled in sections for imaging and dosimetry applications. Such phantoms will be useful for dose verification purposes when commercial phantoms are unavailable for purchase in the specific anatomies of interest.

## 1 INTRODUCTION

Commercial anthropomorphic phantoms such as the RANDO (The Phantom Laboratory Inc, Salem, NY) and ATOM Dosimetry Verification Phantoms (CIRS Inc, Norfolk, VA) have been used for decades to study the performance of imaging systems and to assess the radiation dose received by patients undergoing medical procedures.^1^ These phantoms have human‐like geometry and are composed of materials designed to mimic the photon attenuation and scattering properties of tissue. The tissue‐substitute materials are typically formulated as epoxy resins or polyurethanes with various additives to skew the electron density.^2^, ^3^ Early versions of the RANDO phantom incorporated a real human skeleton, so no two phantoms were exactly alike.^4^

Despite the widespread use of commercial anthropomorphic phantoms, several challenges limit their applications. First, commercial anthropomorphic phantoms only come in a small variety of reference sizes which do not adequately represent all patients. For instance, the RANDO phantoms are only offered in adult male and female varieties. Similarly, the ATOM phantoms are available in adult male and female varieties, as well as four pediatric sizes (newborn, 1‐yr‐old, 5‐yr‐old, and 10‐yr‐old). However, for some research and clinical applications, it is desirable to have phantoms of smaller, larger, or even a patient‐matched size. Second, commercial phantoms are very expensive, with purchase prices as high as ~$25,000. Their high cost can be attributed to manufacturing techniques requiring craftsman‐like skill that have not significantly changed since the late 1970s. Phantoms are produced to order using molding and casting methods which require long manufacturing lead times — one‐of‐a‐kind productions are not cost‐effective with this manufacturing model. Furthermore, a researcher may wish to customize their phantom by drilling additional holes for inserting radiation dosimeters or sources. However, the permanent modification of such an expensive phantom for one‐time use is rarely an attractive option. Lastly, commercial anthropomorphic phantoms have simplified anatomy, often consisting of only three uniform materials representing soft tissue, lung, and bone. Yet, the human body is quite heterogeneous, so existing physical phantoms are not sufficient for applications requiring a high level of anatomical realism.

Given these challenges, it is no surprise that researchers have sought alternatives to physical anthropomorphic phantoms. Computational anthropomorphic phantoms coupled with Monte Carlo radiation transport simulation have proven to be a more flexible and customizable approach. Indeed, unlike their physical counterparts, computational phantoms have evolved much more rapidly.^5^ Today’s computational phantoms come in great variety of sizes (heights and weights), with the most advanced examples containing hundreds of segmented organs or tissues — detail incomparable to any physical phantom available for purchase today.^6^, ^7^ Nonetheless, calculations performed using computational phantoms should be benchmarked against experimental measurements for verification purposes. In most cases, however, the dosimetry data generated using advanced computational phantoms have never been experimentally verified because an equivalent physical phantom does not exist. It is much easier to create a computational version of a physical phantom than it is to bring a computational phantom to life.^8^, ^9^ The ability to custom‐fabricate anthropomorphic phantoms on‐demand for research and other applications would represent a significant breakthrough in the field.

Three‐dimensional (3D) printing is an additive fabrication approach which is ideally suited for creating one‐of‐a‐kind parts, thus offering great promise as a solution for the custom fabrication of physical anthropomorphic phantoms. The technology has been used in medicine for the development of surgical guides, implants, and prosthetics since the early 1990s.^10^ A variety of technologies can be used, such as stereolithography (SLA), fused‐deposition modeling (FDM), selective laser sintering, binder jetting, or material jetting. The printers can range from expensive industrial models to consumer‐grade desktop printers. The consumer‐grade market is currently dominated by thermoplastic extruders (FDM) and prices have dropped significantly over the past decade, bringing the technology to a wider audience and new applications.

Several groups have explored the use of 3Dprinting technology for the fabrication of anthropomorphic phantoms. Alfano et al.^11^ (2003) developed the STEPBRAIN using SLA which featured multiple compartments that could be filled with liquids compatible with positron emission tomography or magnetic resonance imaging. Kim et al.^12^ (2006) developed a Korean male dosimetry phantom using a combination of SLA, molding, and casting. The skeleton of the phantom was printed using a SLA resin with density similar to bone; however, there was no lung‐equivalent material available, so a mold was printed for casting lungs out of urethane foam. Kiarashi et al.^13^ (2015) created breast phantoms using PolyJet technology but found that there was no suitable material available to simulate fat. To overcome this limitation, only the fibroglandular tissue regions were printed, leaving the adipose regions blank to be filled in by a more appropriate material as a postprocessing step. Ehler et al.^14^ (2014) used FDM technology to print a human head phantom out of acrylonitrile butadiene styrene (ABS) plastic. However, they found that ABS plastic tends to warp when printing large, solid parts. For this reason, the head was printed as a hollow shell of ABS that was subsequently filled in with a wax‐based soft tissue simulant — the skull was not considered. Craft and Howell^15^ (2017) created a full‐scale torso phantom using FDM technology. To minimize warping, the authors used polylactic acid (PLA) plastic and a sagittal‐slice design; however, the phantom did not contain bones and the lung regions were left blank. Winslow et al.^16^ (2009) developed a computer‐controlled milling technique that, while not technically 3Dprinting, is also noteworthy because it involved three tissue‐substitute materials. Unfortunately, their method was not fully automated. Anatomical cutouts from slabs of lung, bone, and soft tissue materials were manually assembled, glued, and then sanded to create each transverse slice of the phantoms. Collectively, these efforts have identified several barriers to progress: (a) The small build volume and slow speed of many 3D printers which inhibits the fabrication of human‐size parts; (b) The limited variety of 3D printing materials for simulating tissues with mass densities ranging from 0.25 g/cm^3^ (adult lung) to 1.85 g/cm^3^(bone); and (c) The need to print parts with multiple materials simultaneously. We have yet to identify a 3D printing technology which can fully overcome all these challenges; however, the technology is rapidly evolving with new printers and materials hitting the mass market every year.

In this study, we show how a relatively inexpensive desktop 3D printer can be used to print a full‐scale pediatric torso phantom containing five distinct radiographic regions for computed tomography (CT) imaging applications. This is achieved by combining two different PLA plastics and an anatomical model with a dual extrusion thermoplastic 3D printer. Whereas most thermoplastics have a radiographic density similar to water (~1.0 g cm^‐3^), our method takes advantage of a composite plastic containing iron for simulating bone. At the same time, we spatially vary the infill density of the printed plastic within different anatomical regions of the phantom to achieve more realistic radiographic properties, despite only using two types of plastic. Our approach is unique compared to previously published works in that the fabrication method prints the entire phantom in one build process with very minimal postprocessing and no backfilling of material.

## 2 MATERIALS AND METHODS

An Ultimaker 3 (Ultimaker B.V, Netherlands) FDM 3D printer was used in this study. This consumer‐grade printer (purchase price ~$3.5k) was selected for its open technology framework which allows the user to interface with third‐party slicing software and materials. The printer has a build volume of 21.5 × 21.5 × 20.0 cm and features two independent print cores for printing with up to two different materials during a single build process. The stepping motors for positioning the print nozzles have a nominal accuracy of 12.5 microns within the build‐plane (X‐ and Y‐directions) and 2.5 microns between layers (Z‐direction). The 3D printing was performed using print nozzles of diameter 0.8 mm and a print layer height of 0.4 mm. Previous studies^15^ mostly used 0.4 mm‐diameter nozzles which come standard on many 3D printers; however, we found that a larger nozzle was more time efficient at printing life‐size phantoms. This choice did not result in any significant loss in detail for our imaging application because most clinical CT scanners produce images with pixels ~1 mm in size. The open‐source slicing software Slic3r^17^ was used throughout this study to generate the toolpath (G‐code) files for the 3D printer. We found that this software offered the critical ability to customize print settings to a greater extent compared to Ultimaker Cura, our printer’s manufacturer‐branded freeware.

### 2.A Tissue‐equivalent plastics

Two types of commercially available thermoplastic filament were explored for simulating body tissues: (1) polylactic acid (PLA) (Ultimaker Brand, Dynamism Inc., Chicago, IL) to represent soft tissues and (2) magnetic iron PLA (PLA‐Fe) (ProtoPlant Inc., Vancouver, WA), a composite of PLA and iron powder, to represent bone. Nominal physical properties of these materials are shown in Table 1.

**Table 1:** Characteristics of the thermoplastic filament used in this study.

	Polylactic Acid (PLA)	Magnetic Iron PLA (PLA‐Fe)
Nominal Composition	(C_3_H_4_O_2_)_n_	(C_3_H_4_O_2_)_n_ + Fe *~45% Fe by weight*
Filament Diameter	2.85 mm	2.85 mm
Physical Density^†^	1.23 g cm^‐3^	1.87 g cm^‐3^
Melting Temperature	160 °C	160 °C
Extrusion Temperature	210 °C	180 °C

^†^ measured for an object printed at 100% infill.

**Fig 1:**
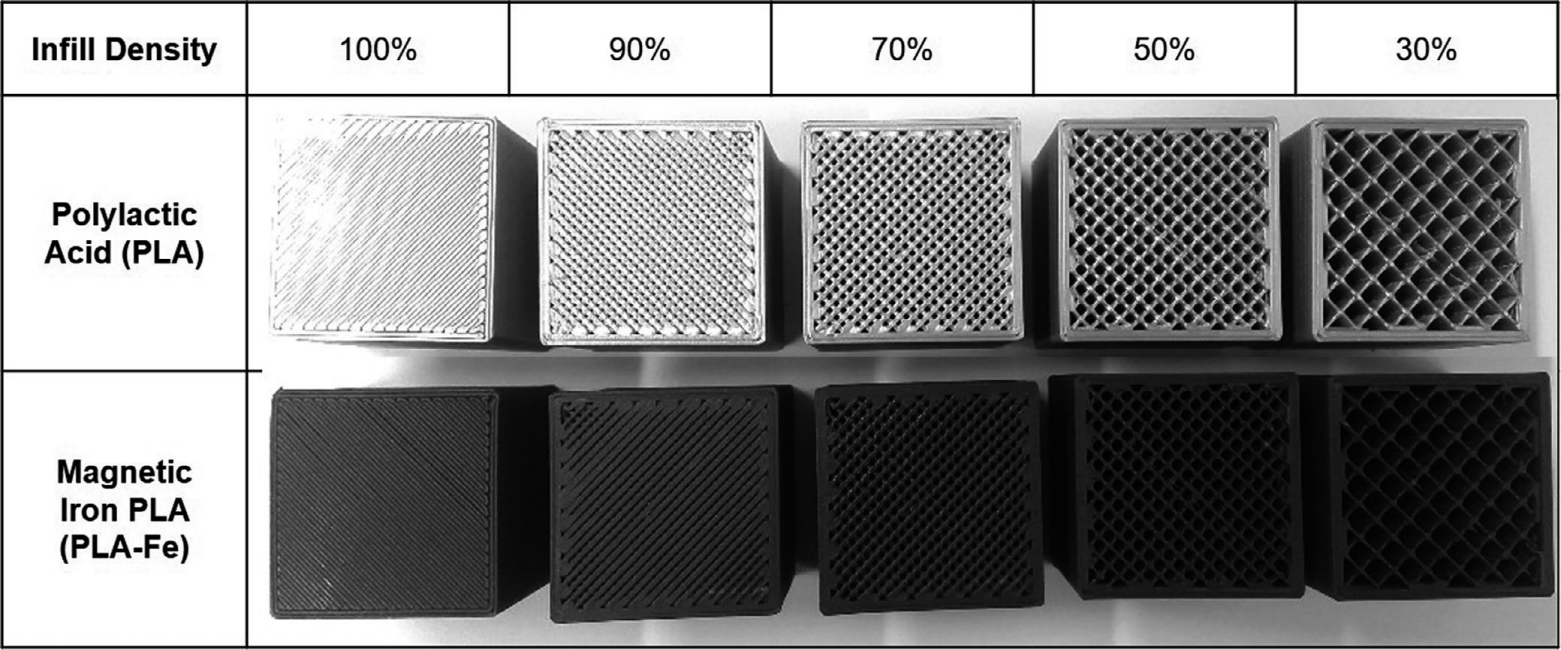
Photographs of the blocks (4 cm side length) which were three‐dimensional‐printed with varying infill density. The top layer of blocks was assigned a thickness of 0 mm to show the internal rectilinear grid of plastic. The blocks printed with 0% infill are hollow and are not shown.

In addition to using two materials, the radiographic density of the printed parts was controlled by varying the infill density setting in Slic3r. The infill density setting is commonly used to speed up the print time and save material by allowing one to reduce the amount of plastic printed on the interior of a part. An infill density setting of 100% produces a solid part, whereas a lower setting introduces small air gaps into the part in a user‐specified infill pattern. The lowest infill density setting is 0% and results in a part which is a hollow shell with a specified wall thickness. Slic3r has several different infill patterns from which the user can select. To limit the scope of our research we focused only on the rectilinear infill pattern with the default infill angle of 45 degrees. The rectilinear infill pattern gives the printed parts an internal geometry similar to that of a parallel hole collimator, with the rectangular holes aligned with the printer build axis (Z‐direction; Fig. 1).

### 2.B Radiographic density calibration

Cubic blocks with side length 4 cm were 3Dprinted out of each plastic (PLA or PLA‐Fe) using different infill density settings ranging between 30% and 100%. The blocks were then CT scanned to generate calibration curves relating the printer infill density settings to the average CT Hounsfield unit (HU) of a printed part. For each plastic a set of up to six blocks were printed for generating the calibration curves. The top layer of each block was assigned a thickness of 0 mm so that the rectilinear infill pattern was visible, as shown in Fig. 1. The exact infill algorithm used by Slic3r was a bit of a “black box,” so to gain better understanding we measured the septa thickness and inter‐septa spacing with a microscope using a magnification of 100X (Dino‐Lite Edge AM4115ZT Digital Microscope, Dunwell Tech Inc., Torrance, CA). The blocks were also weighed to calculate their average physical density. The volume and mass of the walls (1.6 mm thick) were excluded from the density calculation by weighing blocks printed with an infill setting of 0%.

CT imaging of the blocks was performed using a 128‐slice Siemens Biograph mCT scanner (Siemens Medical Solutions, Malvern, PA) located in the Nuclear Medicine Department at the National Institutes of Health’s Clinical Center. The blocks were placed in a row on the bed of the scanner with the build axis (Z‐direction) parallel to the rotational axis of the scanner. Scans were conducted using an abdominal CT protocol (120 kVp, 250 mAs) with 2‐mm thick slices. Images were reconstructed using standard filtered‐back projection with a B40s medium smoothing kernel. The mean and standard deviation of the pixel HU values was calculated for a 20 mm diameter spherical volume of interest (VOI) drawn at the center of each block.

### 2.C Preparation of pediatric torso model

**Fig 2:**
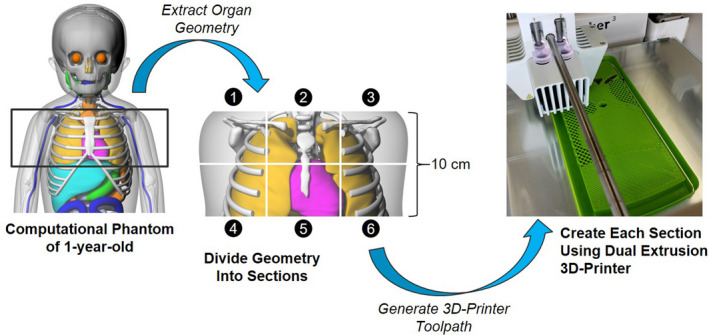
The phantom fabrication process using a dual extrusion three‐dimensional‐printer.

A 10 cm axial cross‐section of a pediatric torso was selected for 3D printing as a demonstration of our phantom fabrication method. The torso geometry used in this study was based on that of a 1‐yr‐old male hybrid computational phantom (height 85 cm, weight 15 kg) picked from the National Cancer Institute’s library of computational phantoms.^6^ The whole‐body computational phantom was originally developed from CT images of a 1‐yr‐old patient and contains over 100 presegmented organs and tissues modeled as either nonuniform rational B‐spline surfaces or a polygon surface mesh (Fig. 2). We selected to use the computational phantom (rather than the original patient CT images) as the source of our geometry to avoid repeating the time‐consuming step of tissue segmentation. Selected organ geometry was imported into the SpaceClaim (SpaceClaim Corporation, Concord, MA) computer‐aided design modeling software to generate the solid geometry needed for 3D printing. A faceted data simplification tool in SpaceClaim called “shrinkwrap” was used for the conversion of some of the complicated polygonal bone surface mesh to solid geometry (e.g., thoracic vertebrae). Boolean operations were performed on the solid geometry to create the different anatomical regions of the phantom. The final torso model consisted of 30 separate anatomical regions including soft tissue remainder, lungs (left and right), heart, esophagus, rib cage (left and right ribs 1 to 10), clavicles (left and right), scapulae (left and right), thoracic vertebrae (one solid object defining thoracic vertebrae T1 to T9). The anatomical assembly was exported in OBJ file format for the development of the printing toolpath (G‐code) in Slic3r.

### 2.D Phantom fabrication

While the build volume of our printer was just large enough to print the phantom as one piece, we opted to divide the torso into six smaller sections (~7 cm wide, 5 cm tall) to be printed separately as shown in Fig. 2. Cylindrical holes were added to the connecting faces of the assembly so that registration pegs could be inserted. The choice to print the phantom in sections was prudent for several reasons. First, this choice helped to minimize the risk of wasted plastic in the event of a printing failure (although this never occurred in this study). Second, our spools of PLA filament only contained 750 g of plastic; therefore, it was necessary to add a new spool of plastic filament before building each section to avoid running out of material mid‐print. Lastly, printing the phantom in small sections helped prevent warping which is a common problem for large, flat prints.^13^ Warping occurs because the 3D‐printed material shrinks as it cools, causing stresses to build inside the cooling part resulting in delamination from the build surface.

Each anatomical region in the torso model was assigned appropriate plastic and infill settings (infill density and wall thickness) in Slic3r to achieve as realistic radiographic properties as possible. The soft tissue and bone regions of the phantom were printed using PLA and PLA‐Fe, respectively. The infill density setting for each anatomical region was selected using the calibration curves created from the printed blocks described in Section 2.B. Our target CT numbers for each anatomical region were selected to match a contrast‐enhanced chest–abdomen–pelvis CT scan of a pediatric patient. The CT images were the same as those used to develop the 1‐yr‐old computational phantom. The heart, soft tissue remainder, and lungs were assigned an infill density of 100%, 94%, and 46%, respectively. These settings were selected in effort to achieve mean target CT values of 183, 60, and −500 HU in the respective regions using normal PLA. For simplicity of the phantom design, all bone regions were assigned an infill density of 50% to achieve a mean target CT number of 1000 HU using PLA‐Fe; this is a typical HU value for cortical (hard) bone, although the average HU value measured over the pediatric patient’s bone structures (comprised of cortical and trabecular bone) was generally smaller. The average CT number for the bone structure examined in this study varied from about 339 to 515 HU, as seen in Table 3. The esophagus was left blank (air). All regions except the heart and lung were assigned a wall thickness of 1.6 mm. The lung regions were assigned a wall thickness of 0 mm to help avoid a seam between adjacent pieces in the transverse plane. The wall thickness of heart was set to 0.8 mm. The top and bottom layers of each region were also assigned a thickness of 0 mm to help avoid a seam between adjacent pieces in the cranial–caudal direction.

Through trial and error, we found that warping of the 3D‐printed phantom pieces could be minimized by using a heated build surface (70 °C), a slow print speed of 16 mm/s, and 200% extrusion width on the first layer. An 8‐mm detachable brim of plastic was added to the first layer of each printed part to help hold the edges down to the build surface. A skirt of PLA plastic surrounded the part, without touching, to reduce cooling airflow. The skirt also served to prime the nozzles and to catch oozing plastic from either nozzle during times of inactive use. To minimize oozing, the inactive extruder temperature was reduced by 20 °C. The entire printer was also placed inside of an enclosed build chamber with temperature control (Model 660, 3DPrintClean, Mountainside, NJ).

### 2.E Phantom verification

CT images of the 3D‐printed torso phantom were acquired using the same scanner settings as described in Section 2.B. The images were compared qualitatively to that of the original 1‐yr‐old patient CT and to CT images of a pediatric torso phantom that was previously purchased by our laboratory (5‐yr‐old ATOM Phantom, CIRS Inc, Norfolk, VA). Quantitative analysis was performed by comparing the mean HU value in various anatomical regions of the 3D‐printed phantom to the target HU values selected in Section 2.D and to that of corresponding regions in the patient and commercial anthropomorphic phantom. Spherical VOIs with diameters 5 to 20 mm were used, as appropriate, depending on the size of the anatomical region. For verification purposes, we also scanned additional PLA blocks printed with an infill density of 46% and 94%. The mean CT number recorded for these blocks was directly compared to what we observed in the lung and soft tissue remainder regions of the 3D‐printed torso.

### 2.F Dose measurements in cylinders

As there is no commercial phantom with the same geometry as our 3D‐printed torso phantom it is challenging to do a meaningful experimental dosimetry comparison to some already recognized standard. Ultimately such a dose comparison might be performed through Monte Carlo simulation involving our computational phantom, but such an effort is beyond the scope of the current work. Instead, dose measurements were performed in a series of 3D‐printed cylindrical holders (14 mm diameter, 25 mm length) with infill densities of 35%, 50%, 80%, 90%, 95%, and 100%. Each cylinder had slot for inserting an Al_2_O_3_‐based optically stimulated luminescent dosimeter (OSLD) of size 10 mm × 10 mm × 2 mm (screened nanoDots, Landauer, Glenwood, IL). The cylinders with the OSLDs inserted were placed on the bed of the CT scanner. The OSLD stored signal was read with a microSTARii reader (Landauer, Glenwood, IL) before and after a single CT body scan (120 kVp, 250 mAs).

## 3 RESULTS

### 3.A Radiographic density calibration

**Fig 3:**
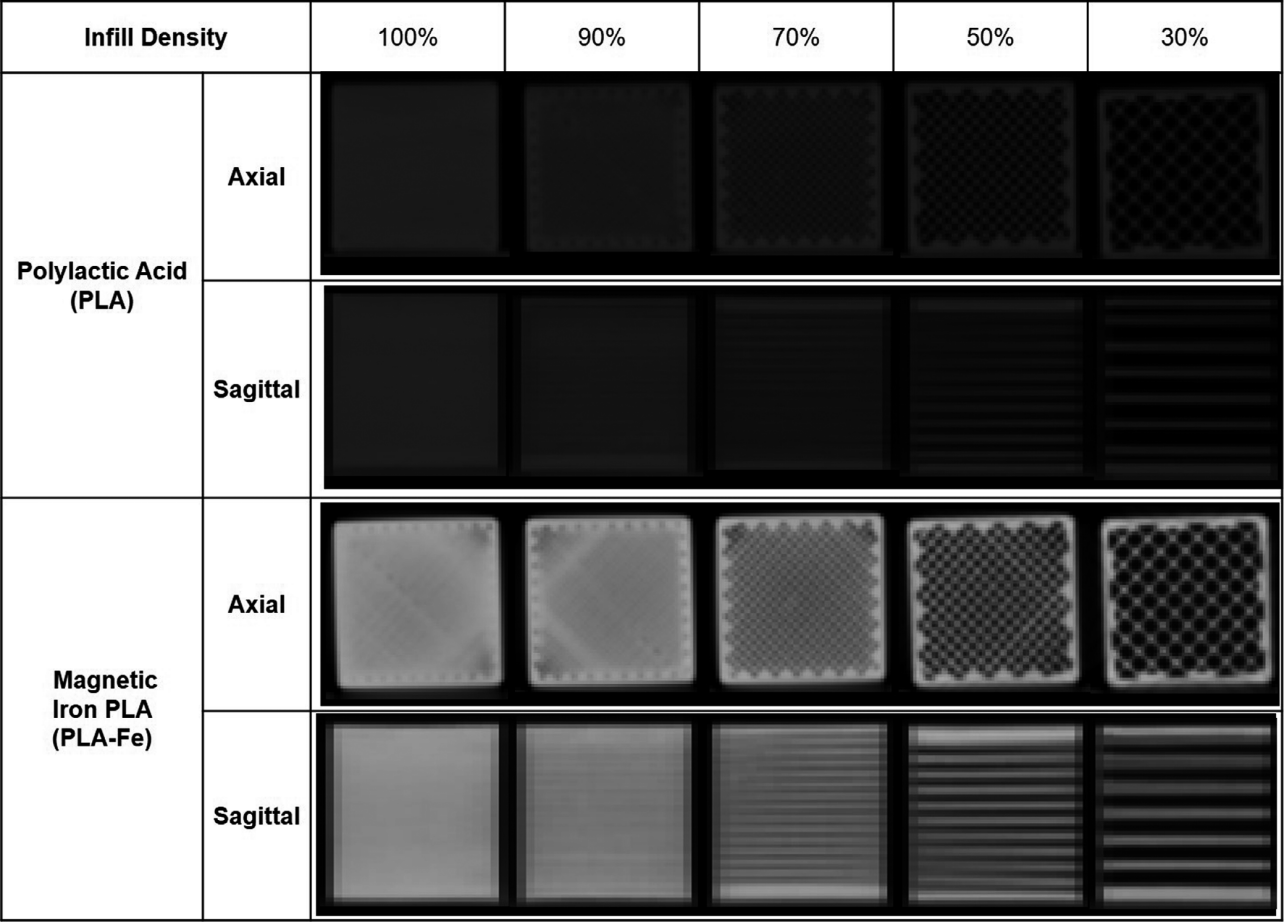
Sagittal and axial computed tomography images for the three‐dimensional ‐printed blocks using polylactic acid (PLA) and PLA‐Fe plastics with different infill densities.

Axial and sagittal CT images of the 3D‐printed blocks are shown in Fig. 3. The air gaps were not clearly visible within the resolution of the CT (pixel size 0.5859 × 0.5859 × 2.0 mm) for the blocks with infill density 90% and larger. The holes, however, were visible in the images of the blocks with infill density 70% and smaller. Measurements of the septa thickness and inter‐septa spacing can be found in Table 2. As expected, the inter‐septa spacing decreased with increasing infill density. The septa thickness was ~0.8 mm and was relatively constant for infill densities up to 50%. However, a septa thickness of 0.858 mm and 1.019 mm was measured for the PLA blocks with infill densities of 70% and 90%, respectively. These results suggested that the Slic3r infill algorithm is controlling both the thickness and separation of the septa when the infill density is varied. Minor variations between the PLA and PLA‐Fe septa thickness and inter‐septa spacing were observed, even though the printing toolpath files (G‐code) were the same except for the extrusion temperature for PLA‐Fe which was 30 °C cooler than for PLA.

**Table 2:** Measured properties of the three‐dimensional (3D)‐printed blocks using polylactic acid (PLA) and PLA‐Fe

Infill Density	Polylactic Acid (PLA) Blocks				Magnetic Iron PLA (PLA‐Fe) Blocks			
	Septa Thickness (mm)	Inter‐Septa Spacing (mm)	Average Density (g cm^‐3^)	Mean HU	Septa Thickness (mm)	Inter‐Septa Spacing (mm)	Average Density (g cm^‐3^)	Mean HU
*For Calibration*								
100%^†^	1.089 (0.034)	~0	1.23	183 (8)	1.129 (0.023)	0.062 (0.015)	1.87	2449 (45)
90%	1.019 (0.018)	0.284 (0.022)	1.04	−21 (19)	1.098 (0.037)	0.226 (0.037)	1.69	2153 (50)
70%	0.858 (0.010)	0.870 (0.015)	0.83	−228 (75)	0.914 (0.032)	0.775 (0.019)	1.40	1558 (152)
50%	0.740 (0.013)	1.643 (0.024)	0.62	−446 (187)	0.764 (0.040)	1.636 (0.065)	0.98	901 (421)
40%	0.709 (0.013)	2.292 (0.027)	0.48	−545 (425)	–	–	–	–
30%	0.712 (0.018)	3.225 (0.023)	0.38	−661 (256)	0.775 (0.023)	3.404 (0.030)	0.63	252 (675)
*For Verification*								
94%	1.009 (0.024)	0.261 (0.027)	1.06	−1 (58)	–	–	–	–
46%	0.730 (0.024)	1.859 (0.021)	0.54	−482 (406)	–	–	–	–

Note:

The standard deviation of measured values is shown in parentheses. Septa thickness and spacing were measured using a Dino‐Lite Edge AM4115ZT Digital Microscope (Dunwell Tech Inc., Torrance, CA) at a magnification of 100X.

^†^ For the PLA block printed at 100% no line separation was clearly visible so septa and inter‐septa spacing were difficult to measure.

**Fig 4:**
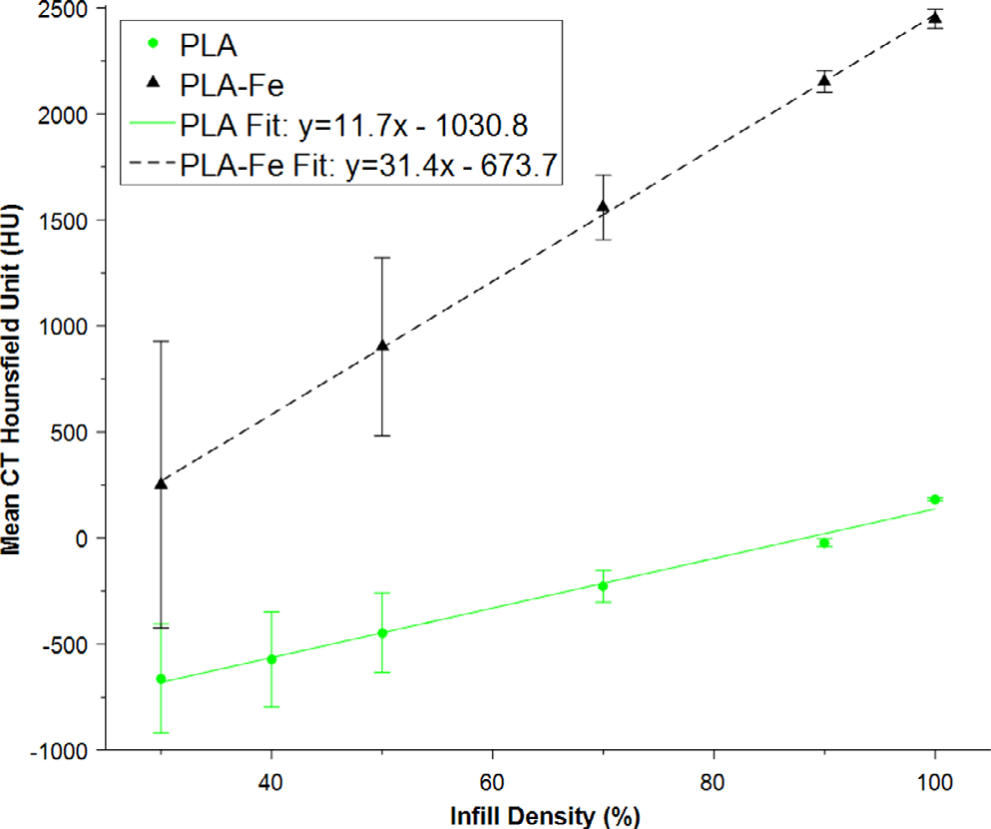
Calibration lines relating the printer infill density to the measured computed tomography Hounsfield unit (120 kVp scan) for the blocks printed out of each type of plastic. The blocks were printed using a 0.8 mm nozzle with 0.4 mm thick layers and a rectilinear infill pattern. The data points (error bars) represent the mean (standard deviation) of the pixel HU values recorded for a 2 cm diameter spherical ROI at the center of each block.

Table 2 shows the measured physical density of the interior of each block as well as the mean (standard deviation) CT number recorded for a 20 mm diameter spherical ROI placed at the center of each block. The CT numbers of the PLA blocks varied from 183 HU to −661 HU as the infill density was changed from 100% to 30%, a range sufficient for simulating most soft tissue in the body, including lung. The physical density of the PLA blocks varied from 1.23 g cm^−3^ to 0.39 g cm^−3^. Similarly, for the PLA‐Fe blocks, the radiological density varied from 2449 HU to 252 HU as the infill density was changed from 100% to 30%, covering the typical range of CT numbers expected for cortical bone. The physical density for these blocks varied from 1.87 g cm^−3^ to 0.63 g cm^−3^. A strong linear relationship was found between the printer infill density setting and mean CT number measured for each block as shown in Fig. 4. It is important to note, however, that the standard deviation of the HU values within each block increased with decreasing infill density, as expected, because of the material heterogeneity; with decreasing infill density the distribution of pixel values becomes increasingly double‐peaked. Lines of best fit were generated for purposes of interpolation. We also compared the measured HU values for blocks of 100% PLA with different colors (silver, green, magenta) and found some small systematic differences in CT number which were not anticipated (measured differences <40 HU).

### 3.B Phantom fabrication

**Fig 5:**
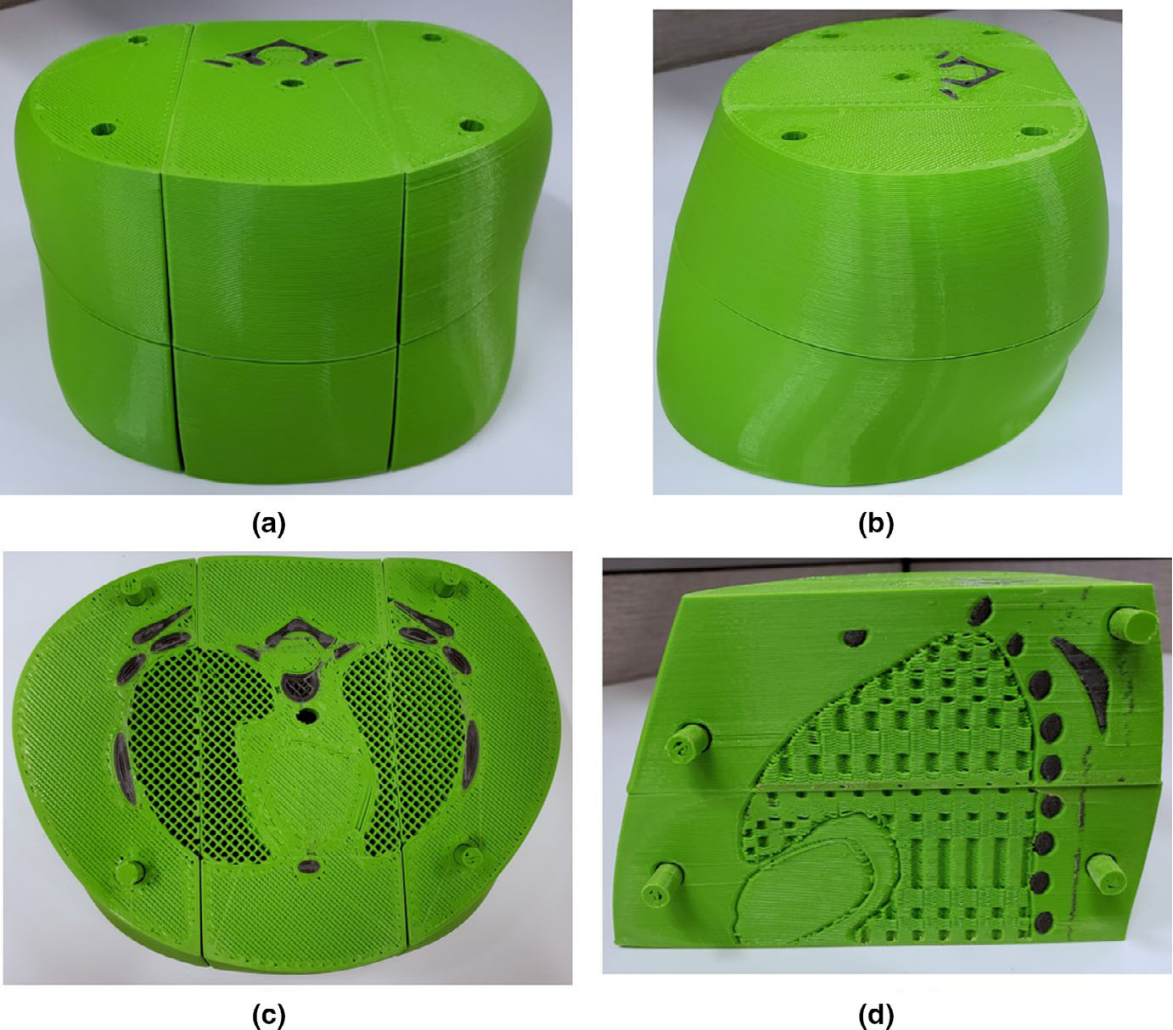
Photographs of the three‐dimensional ‐printed pediatric torso phantom. (a) The fully assembled phantom. (b) Side view of the fully assembled phantom. (c) Axial view of the lower phantom sections (top sections removed). (c) Sagittal view with two left sections removed.

The six pieces of the torso phantom were printed over the course of a week (Fig. 5). Each section took approximately 15 to 24 h to print and printed correctly (without printing failure) on first attempt. In total, approximately 300 meters of 2.85‐mm diameter plastic filament were used with an estimated material cost of $160. The total weight of the phantom was 2.4 kg. Postprocessing involved lightly sanding the adjoining faces of the phantom sections with a belt sander until they sat flush against each other. The sections were then connected using cylindrical registration pegs (7 mm diameter, 19 mm length) which were printed as part of a separate build process.

### 3.C Phantom verification

**Fig 6:**
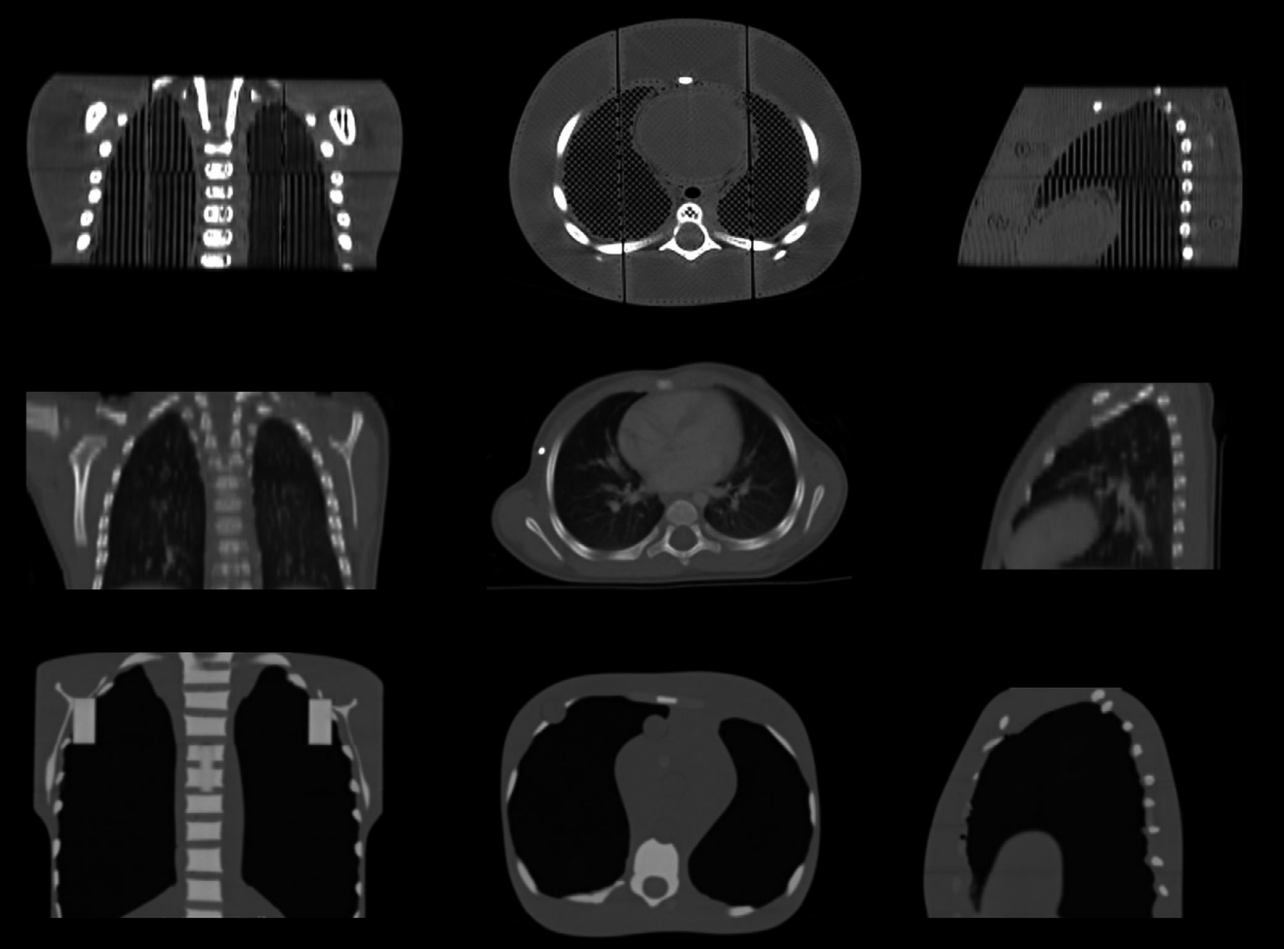
Comparison of computed tomography (CT) scans for the three‐dimensional ‐printed phantom (top row), 1‐yr‐old patient CT used as the source geometry (middle row), and a commercial CIRS 5‐yr‐old phantom (bottom row). Coronal, axial, and sagittal views of the torso are shown in the left, middle, and right columns, respectively. Images are shown to scale using same grayscale settings with level (center) of 300 HU and window of 2400 HU.

**Fig 7:**
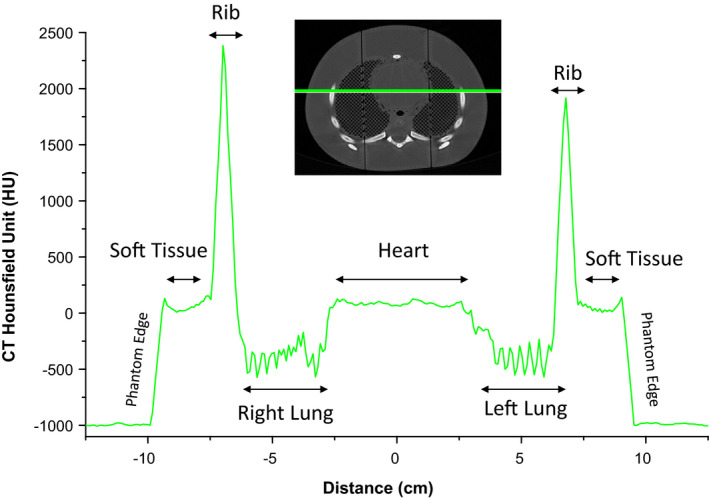
Measured HU values along a right‐left line segment in the torso. To reduce noise the mean HU for a 10 mm sliding window is shown.

CT images of the 3D‐printed phantom are shown in Fig. 6 along with those of the 1‐yr‐old patient and CIRS 5‐yr‐old phantom for comparison purposes. The 1‐yr‐old phantom anatomy (middle row) shows a high degree of similarity to that of the 3D‐printed phantom (top row) because it was used as the basis for creating the computational phantom from which the geometry for 3D printing was derived. One notable difference is in the outer body contour of the phantom; when the computational phantom was created, an adjustment was made to outer body contour to match the weight of the phantom to reference person characteristics. The positioning of the clavicles is also different; the patient has arms raised as is typical during CT scanning whereas the computational phantom’s posture was altered to have arms at the side. Despite these systematic differences, our results demonstrate the remarkable capability of 3Dprinting to capture individualized anatomy with a high degree of fidelity. Furthermore, it can be observed that the CIRS phantom has uniform density within the lung and bone regions, whereas the 3D‐printed phantom has a significant amount of texture which more closely resembles that seen in patients. A profile of the measured HU values measured laterally through the phantom is shown in Fig. 7, which was created by averaging over a 10 mm sliding widow to reduce noise. The profile for the 5‐yr‐old commercial phantom (not shown) is qualitatively similar.

A quantitative comparison of CT Hounsfield units in various anatomical regions was performed and the results are shown in Table 3. The infill density settings for the various regions of the 3D‐printed phantom were selected in effort to achieve target CT numbers of 183, 60, −500, 1000 HU in the heart, soft tissue remainder, lungs, and bone regions, respectively. Analysis of the CT images of the 3D‐printed phantom showed that we could achieve these target CT numbers to within 100 HU or 200 HU in the case of soft tissues and bone, respectively. The mean (standard deviation) of the CT numbers in the heart, soft tissue remainder, right lung, and vertebrae body of the 3D‐printed phantom were 94 (46), 31 (79), −417 (434), and 1180 (1107) respectively. These values were compared to that of the 3D‐printed blocks printed with the same infill density settings used in these regions to check for differences from the calibration conditions. The mean (standard) deviation of CT numbers for the PLA blocks printed at 100% (heart), 94% (soft tissue remainder), and 46% (lung) infill were 183 (8), −1 (58), and −482 (406), respectively. For the PLA‐Fe block printed at 50% (bone) these values were 901 (421) HU.

**Table 3:** Measured HU values for various anatomical regions in the three‐dimensional (3D)‐printed phantom (this study), 1‐yr‐old patient computed tomography (CT) (anatomy used as source geometry), and a commercial CIRS 5‐yr‐old phantom

Anatomical Region	Measured Mean (Standard Deviation) CT Hounsfield Unit		
	3D‐printed Phantom	1‐year‐old Patient	CIRS 5‐year‐old Phantom
Heart	94 (46)	174 (38)	16 (33)
Soft Tissue Remainder^†^	31 (79)	–	19 (66)
Vertebrae Body	1180 (1107)	339 (75)	729 (52)
Scapula	1290 (1248)	373 (104)	724 (59)
Clavicle	1190 (632)	515 (337)	739 (122)
Sternum	1646 (986)	440 (83)	703 (166)
Right Lung	−417 (434)	−538 (83)	−793 (10)
Air Cavity^‡^	−904 (88)	−915 (155)	–

^†^ Soft tissue remainder of patient image not reported because heterogenous and not clearly defined.

^‡^ Esophagus (3D‐printed phantom) or Trachea (patient). No air cavity in CIRS phantom.

### 3.D Dose measurements in cylinders

**Fig 8:**
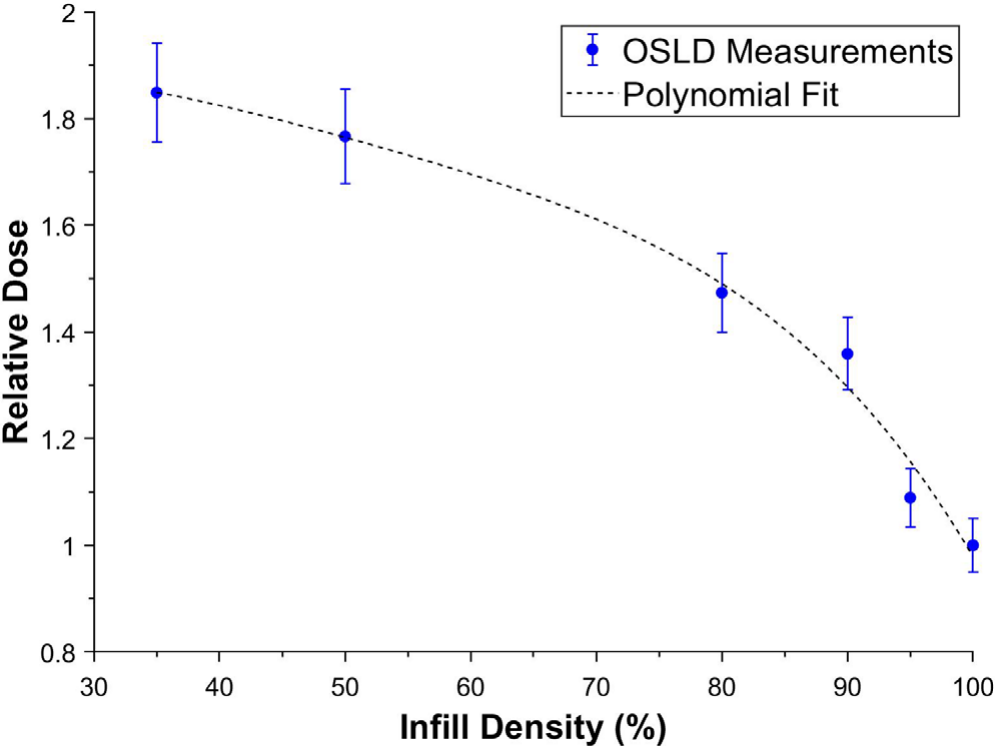
The optically stimulated luminescent dosimeter dose response increases with decreasing infill density.

As the microSTARii reader was not calibrated for measuring absolute dose for CT x‐ray beams, the OSLD readings were normalized to that of the OSLD in the cylinder with 100% infill (solid). Figure 8 shows that the OSLD response increased nonlinearly with decreasing infill density as expected. These results demonstrate that the dose reading within a 3D‐printed object can be modulated by spatially varying the infill density.

## 4 DISCUSSION

While commercial phantoms come in limited sizes, 3D printing has the advantage of allowing the creation of custom size phantoms for imaging and dose verification. Such an approach becomes especially useful to fill the gap when an appropriately sized commercial physical phantom is not available for purchase, such as in the case of premature infants. Infants in neonatal intensive care units sometimes receive multiple radiograpic procedures; however, as these patients are often smaller than typical infant it can be challenging to choose the optimal radiation technique to minimize patient exposure. Existing literature on this topic describe doses to premature infants mostly in terms of machine output parameters, not organ absorbed dose, in part because no premature infant phantom exists for making direct measurements.^18^

An achievement of our work is the creation of a custom‐sized physical anthropomorphic phantom with five distinct radiographic regions (heart, soft tissue remainder, lung, bone, and air cavity), despite using only two types of plastic. The strength of our phantom fabrication method is that it is essentially automatic — all the design processes are performed in a few hours on the computer and the phantom is printed in a single build process with the click of a button with no backfilling of materials. There are, however, several key limitations which are described below.

The differences between our targeted (original patient CT) and actual CT numbers measured in our 3D‐printed phantom can be explained by limitations in our calibration method. First, it should be noted that the calibration blocks were measured in‐air, whereas the measurements within the phantom are affected by beam hardening and scatter to a greater extent. Second, the calibration block measurements were performed using a large spherical VOI placed at the center of the blocks and far from the walls; however, the measurement conditions in the phantom were sometimes different, particularly in the case of bone. The bone structures in the phantom were small and thin, making it hard to identify a suitable internal region large enough for averaging without interference from the solid wall perimeters. Third, the calibration curves (Fig. 4 **)** are a function of the CT tube voltage (kVp), image resolution, and image reconstruction algorithm; these were kept constant in our work, but limit the applicability of the calibration curves we generated. Lastly, we did observe some systematic differences in the way the printer laid down plastic when printing the phantom compared to the calibration blocks. For instance, the septa thickness (inter‐septa spacing) in the heart region of the 3D‐printed phantom was 1.089 ± 0.034 mm (0.116 ± 0.051 mm), whereas there was no visible spacing in the solid PLA block. We compared line spacing in the toolpath files (G‐code) for the heart and solid block generated by the Slic3r software and found them to be the same; therefore, the observed differences are attributed to inconsistencies in the printer. Despite our best efforts to calibrate the printer, the printing process was not as reproducible as desired. The third‐party Slic3r slicing software used in this study offered the critical ability to assign the infill density to different regions in an assembly of parts; however, it did not have the capability to directly define the infill line thickness and spacing as we would have liked. As future work, it will be important to seek ways to more reliably predict the CT number of our 3D‐printed objects. One such way might be to incorporate the grid septa directly into our 3D model.

Another key constraint on our method had to do with the 3Dprinting materials we used. The PLA‐Fe used in this study had a CT number which was too large when printed solid to represent pediatric bone (2449 HU vs 350 HU). Similarly, when printed solid, the normal PLA had a CT number too large to represent lung (183 HU vs −500 HU). To overcome this limitation we reduced the printer infill density within these anatomical regions. The obvious drawback of this approach is that it results in a phantom which is not comprised of a solid material, creating an anisotropy in radiation attenuation depending on the orientation of the hollow channels with respect to the radiation source. This was overcome, to some extent, in the present work by exploiting the axial symmetry of the CT scanner; however, this approach will clearly not work for all applications. Therefore, future research efforts should focus on creating customized 3Dprinting materials to serve as better tissue simulants when printed solid, particularly for bone and lung. For instance, the PLA‐Fe used in this study might be improved as a bone simulant by reducing the amount of iron in the filament. The creation of 3Dprinting filament to represent lung poses a much bigger challenge because nearly all 3Dprinting materials on the market have a physical density between 0.9 and 1.2 g cm^−3^. Our 3D printer, however, featured only two print nozzles, and printing two materials solid would not provide enough variation in radiographic density for a realistic anthropomorphic phantom; one would need at least three nozzles to print a phantom with three materials to represent lung, bone, and soft tissue. Another option might be to use a printer design which can efficiently switch between three materials. Lastly, it should be noted that we could not vary the infill density continuously in our phantom. Only one infill density setting could be assigned to each anatomical region. Future FDM printers may be able to vary materials continuously through appropriate mixing of an array of plastics.

## 5 CONCLUSION

In this study we demonstrated how an inexpensive desktop 3D printer can be used to print a full‐scale pediatric torso phantom containing five distinct radiographic regions for computed tomography (CT) imaging applications despite using only two types of plastic. While our method has key limitations, our results show that the creation of patient‐specific imaging phantoms of comparable quality to commercial phantoms are possible with existing 3Dprinting technology. In principle, our methods can be improved by using a customized 3D printer, slicing software, and materials; however, such efforts were beyond the scope of this study, as our intent was to use off‐the‐shelf supplies. With more work along these lines of research, we expect that the ability to create patient‐specific phantoms on‐demand will soon become a reality, and this will have important research and clinical applications throughout the field of medical physics.

## CONFLICT OF INTEREST

The authors declare no conflict of interest.
